# Mn-Modified ZnO Nanoflakes for Optimal Photoelectrochemical Performance Under Visible Light: Experimental Design and Theoretical Rationalization

**DOI:** 10.1021/acs.jpclett.3c02730

**Published:** 2023-10-20

**Authors:** Abinash Das, Dongyu Liu, Riu Riu Wary, Andrey S. Vasenko, Oleg V. Prezhdo, Ranjith G. Nair

**Affiliations:** † HSE University, 101000 Moscow, Russia; ‡ PSG Institute of Advanced Studies, Coimbatore, Tamil Nadu 641004, India; § Solar Energy Materials Research & Testing Laboratory (SMaRT lab), Department of Physics, National Institute of Technology Silchar, Silchar, Assam 788010, India; ∥ Donostia International Physics Center (DIPC), 20018 San Sebastián-Donostia, Euskadi, Spain; ⊥ Department of Chemistry, University of Southern California, Los Angeles, California 90089, United States; # Department of Physics & Astronomy, University of Southern California, Los Angeles, California 90089, United States

## Abstract

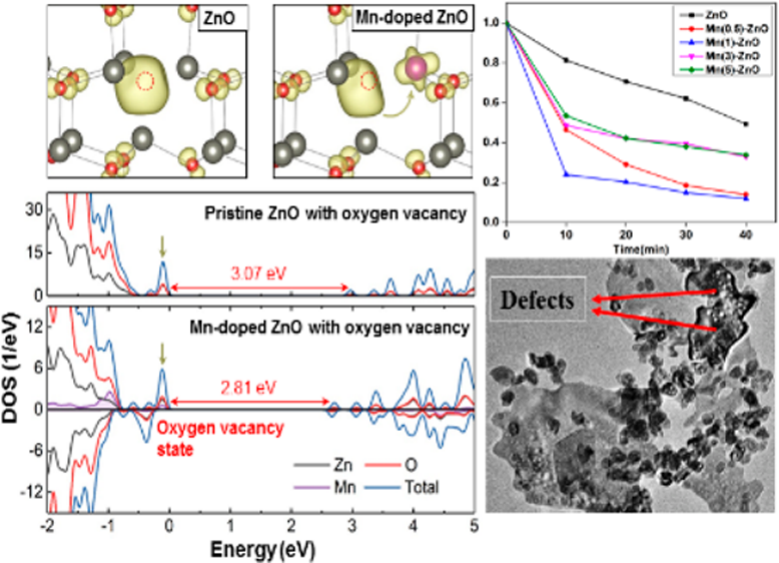

Doping of zinc oxide (ZnO) with manganese (Mn) tunes midbandgap states of ZnO to enhance its optical properties and makes it into an efficient photoactive material for photoelectrochemical water splitting, waste removal from water, and other applications. We demonstrate that ZnO modified with 1 at. % Mn exhibits the best performance, as rationalized by experimental, structural, and optical characterization and theoretical analysis. ZnO doped with the optimal Mn content possesses improved light absorption in the visible region and minimizes charge carrier recombination. The doping is substitutional and creates midgap states near the valence band. Mn atoms break localized charge traps at oxygen vacancy sites and eliminate photoluminescence peaks associated with oxygen vacancies. The optimal performance of Mn-modified ZnO is demonstrated with the photodegradation of Congo red and photoelectrochemical water splitting.

Photocatalytic and photoelectrochemical (PEC) approaches are being considered as potential technologies for the cleanest and most efficient way of solar-driven renewable energy conversion.^1−5^ Limitations of most current photoactive materials in achieving high efficiencies drive active research toward development of better photocatalysts. Material response to visible light illumination and lifetimes of photogenerated charge carriers can significantly influence the performance. For instance, zinc oxide (ZnO) is a stable and inexpensive photoactive metal oxide used in many energy-related applications, such as water splitting and removal of organic dyes and other waste, because of suitable band positions and uniform morphologies.^6−9^ However, pristine ZnO utilizes only the ultraviolet portion of the solar spectrum, limiting its overall performance.^10−13^ Fast electron–hole recombination, facilitated by ZnO defects, further reduces its performance. At the same time, suitable nanostructured morphologies provide large surface area for light harvesting and photocatalytic activity, while also maintaining efficient pathways for charge and molecule transport and photocurrent generation.^14−16^ ZnO doping is promising for utilizing the visible-light spectrum and electron–hole separation.^17−20^ It is essential to determine appropriate dopants and their optimal presence in the ZnO lattice in order to optimize the optical and electronic properties of ZnO. In this context, rare-earth elements are excellent choices as dopants, due to their special electronic shell structure and optical features at different energy levels.^21−25^ It is expected that Mn with a comparable ionic radius to that of Zn can be doped inside ZnO nanostructures to enhance the optical properties under visible illumination.^26,27^ Gao et al. demonstrated how Mn ions could easily replace Zn ions due to the comparable ionic radii.^28^ Studies on Mn-doped ZnO substantiate the fact that the interaction between Mn and ZnO can influence the electronic properties and bandgap of the material. An optimal dopant fraction in executing better performance through improved photoactivity needs to be determined. It is anticipated that electron–hole pairs can be separated more efficiently in Mn-doped ZnO than in undoped ZnO due to vacancy levels, which are formed in such a way that the photogenerated electrons and holes can simultaneously participate in reactions without being accumulated on the semiconductor surface.^29^ The charge separation extends the charge carrier lifetime, improving the photoactivity. Accordingly, it is essential to elucidate the electronic structure in doped environments for the rational design of the material for visible-light photoactivity. In this context, density functional theory (DFT) simulations have emerged as a convenient approach to complement synthesis and experimental characterization.

In this work, Mn-doped ZnO was prepared using a simple coprecipitation method and applied to photoelectrochemical water splitting and photodegradation of Congo red (CR). ZnO doped with Mn at different concentrations was systematically characterized using experimental and theoretical tools to gain detailed information about the role of doping in improved performance. The effects of the optimal dopant fraction on the bandgap, charge carrier dynamics, and photoactivity of ZnO were established.

Pristine and Mn-doped ZnO samples were prepared using a simple coprecipitation method. Zinc nitrate hexahydarte (Zn(NO_3_)_2_·6H_2_O), sodium hydroxide (NaOH) and manganese(II) sulfate (MnSO_4_·H_2_O) were used as starting precursors. Accordingly, for the synthesis of Mn-doped ZnO at different atomic percentages (0.5, 1.0, 3.0, and 5.0 at. %), appropriate amounts of zinc nitrate and manganese(II) sulfate were mixed in 100 mL of distilled water under stirring for 10 min. A freshly prepared aqueous NaOH solution (1 M) was added dropwise until the pH of the solution reached 10, which turned the solution into a white gelatinous precipitate. The white precipitate was filtered and washed multiple times with distilled water and ethanol to remove impurities. The samples were transferred inside an oven operating at 150 ^◦^C for 12 h, followed by calcination at 400 ^◦^C for 1 h in a muffle furnace. Undoped ZnO was also prepared for comparison using the same approach but without the use of manganese(II) sulfate. Correspondingly, the produced samples were named ZnO, Mn(0.5)-ZnO, Mn(1)-ZnO, Mn(3)-ZnO, and Mn(5)-ZnO. The physicochemical properties of the materials were studied using different characterization methods, and the details are provided in the Supporting Information. The details of the photocatalytic tests and linear sweep voltammograms (LSV) are also given in the Supporting Information.

The DFT calculations were carried out using the Vienna *Ab initio* Simulation Package (VASP).^30−33^ The projector augmented-wave (PAW) method was adopted to describe the electron–ion interactions.^34,35^ The Perdew–Burke–Ernzerhof (PBE) functional^36^ was used for geometry optimization. The DFT+U correction was applied to Mn 3d electrons with an effective *U* of 5 eV.^37,38^ The electronic structure was calculated with the PBE0 hybrid functional to get a more accurate bandgap.^39^ The cutoff energy of the planewave basis was set to 500 eV, and a Gaussian smearing with σ = 0.05 eV was used. ZnO bulk was represented by a 3 × 3 × 2 supercell containing 72 atoms. We constructed the oxygen vacancy and Mn-doped systems by removing an O atom and replacing a Zn atom with a Mn atom, respectively. A 2 × 2 × 2 *k*-point mesh was employed to sample the Brillouin zone. The dispersion interactions were included using the DFT-D3 model.^40,41^ The structures were visualized using the VESTA software package.^42^

Figure 1a shows the structural phases of the samples analyzed by XRD. The obtained diffraction peaks and identified planes match those of the hexagonal wurtzite phase of ZnO (JCPDS 36-1451). The doping of Mn into the ZnO lattice did not result in formation of a new crystal phase, confirming successful incorporation of the foreign atoms into the ZnO lattice, made possible due to the smaller ionic radius of Mn compared to Zn.^28^ A slight red shift in the XRD peak, as compared to the pristine ZnO, was observed once the doping reached the optimal limit (1 at. %). This minimal shift in 2θ without formation of any additional phases suggests that Mn doping occurs in ZnO crystal sites. The mean crystallite size of the samples was estimated from the XRD data using Scherrer’s formula, as shown in Table 1. In addition, the microstructure of Mn-doped ZnO (1 at. %) was analyzed from the TEM images. Nonuniform flakes-like structures were observed. As shown in Figure 1b, the white dots present on the surface of the flakes correspond to formation of defects (shown by the arrows and inset in Figure 1b), which is consistent with the results of Gupta et al.^43^ The observed defects correspond to oxygen vacancies formed on the catalyst surface. This can be further confirmed by photoluminescence (PL) and XPS spectroscopy. The high-resolution TEM (HRTEM) image in Figure 1c shows the lattice spacing of 0.24 nm, consistent with the *d*-spacing of the (101) diffraction plane of the hexagonal wurtzite phase of ZnO. A clear, bright diffraction ring corresponding to the (102), (112), and (201) planes is marked in the selected area diffraction (SAED) pattern in Figure 1d, confirming the crystalline nature of the samples, as supported by the XRD pattern.

**Figure 1:**
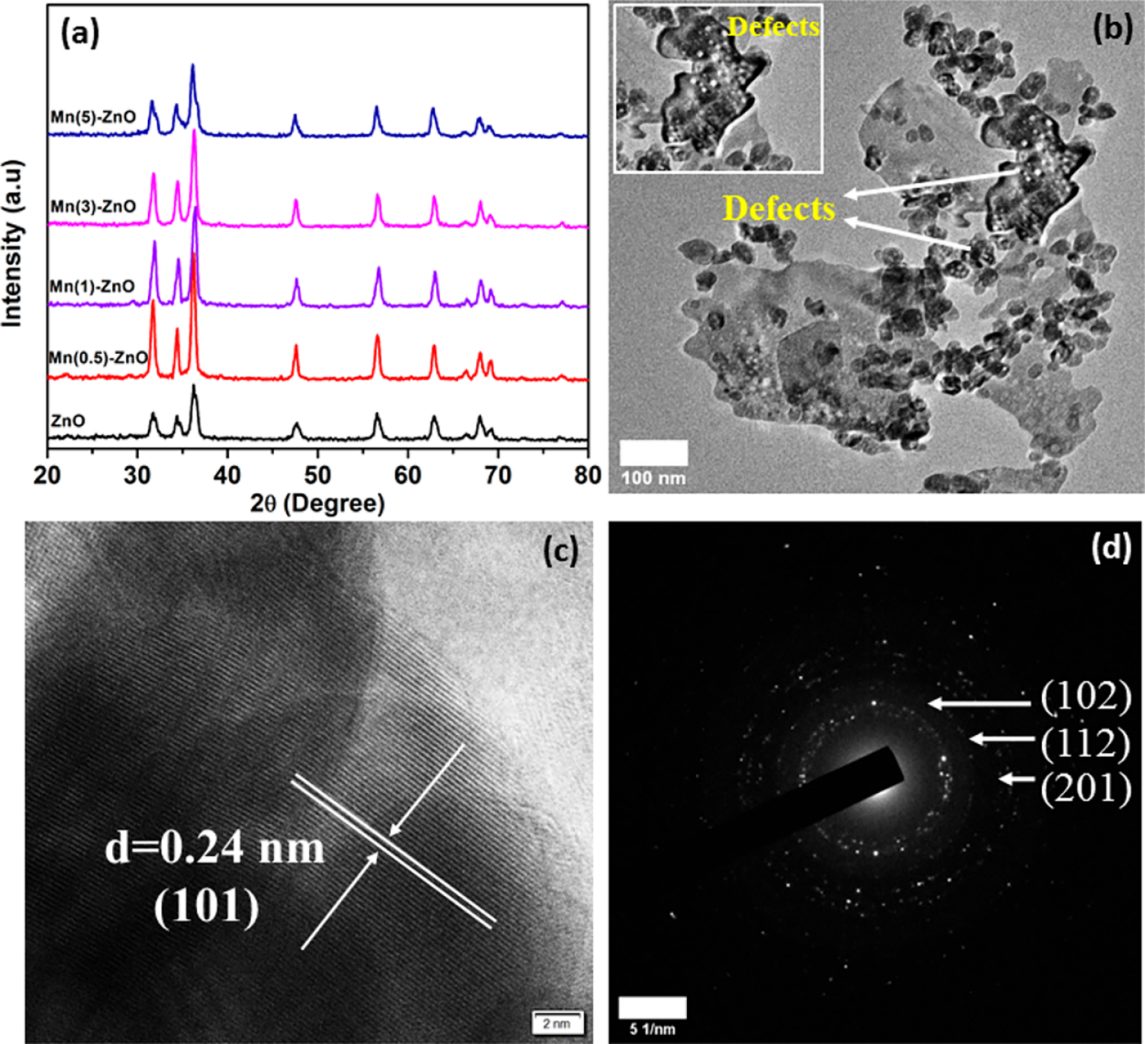
(a) XRD spectra of the samples. (b) TEM image showing the existence of defects after Mn doping, (c) HRTEM image and (d) SAED pattern of Mn(1)-ZnO.

**Table 1:** Crystallite Size and Other Parameters of the Samples

Sample	Crystallite Size (nm)	Bandgap (eV)	Rate constant (min^–1^)	Photonic efficiency (%, 10^–3^)	Photocurrent (mA)
ZnO	15.04	3.19	0.017	0.60	0.14
Mn(0.5)-ZnO	20.46	3.02	0.053	1.58	0.20
Mn(1)-ZnO	19.33	2.78	0.062	1.69	0.84
Mn(3)-ZnO	19.10	2.92	0.032	1.07	0.52
Mn(5)-ZnO	20.73	2.94	0.031	1.03	0.44

The photoactivity of the catalyst is mainly dependent on the surface morphology of the materials. The SEM image provided in Figure SI 1 displays formation of 2D nanoflake-like structures for both undoped and doped ZnO, which is in accordance with the TEM images. The SEM image indicates that the agglomeration of ZnO nanoflakes consists of larger particles. The SEM image of doped samples shows the formation of smaller particles; this may be due to the addition of Mn as a dopant. The content of Mn in ZnO is increased with doping, which thereby obstructs the growth of ZnO nanoflakes and creates an irregular structure at a higher doping content. Similarly, the EDXA spectrum (Figure SI 2) identifies that the doped samples contain Zn, O, and Mn elements without involving significant peaks of other elements. Thus, EDXA confirms the successful incorporation of Mn into the ZnO lattice.

The optical absorption of the samples was examined by using UV-DRS analyses. The bandgap values were calculated from Eg = 1240/λ, where “λ” is the threshold wavelength of the material. As depicted in Figure 2a, the doped samples showed an enhanced shift in absorption toward the higher wavelength region attributable to the insertion of Mn inside the ZnO lattice.^44^ There is a predominant red shift toward the visible region with an increase of Mn content in ZnO, The reduction in absorption is observed with a further increase in Mn doping beyond 1 at. %, and it is attributed to the doping limit of ZnO. Thus, the optimal Mn content for the doped samples may play an important role in improving the photoactivity of the samples. The PL study of the samples was also performed to understand the dynamics of the photogenerated charge carriers, as shown in Figure 2b. The rate of charge carrier recombination can be correlated to the PL emission intensity resulting from the defect states, which eventually determines the improved PEC and photocatalytic activity of the samples.^45,46^ The PL emission spectra revealed that the incorporation of the optimal Mn content inside the ZnO lattice could reduce the electron–hole recombination rate significantly, which is confirmed by the low PL intensity of Mn(1)-ZnO compared to the remaining doped and undoped ZnO. The peaks that appeared in the PL emission spectra originate from the native defect states of ZnO, such as near-band emission (NBE) in the UV region and other peaks located in the visible region assigned to the zinc interstitial (Zn_i_), zinc vacancy (V_Zn_), and oxygen vacancy (V_O_) of the samples. The peaks corresponding to oxygen vacancy found in the near 550 nm range are anticipated to have a crucial impact on the optical properties of the samples,^6,47^ which is further examined using XPS in the later section of the work.

**Figure 2:**
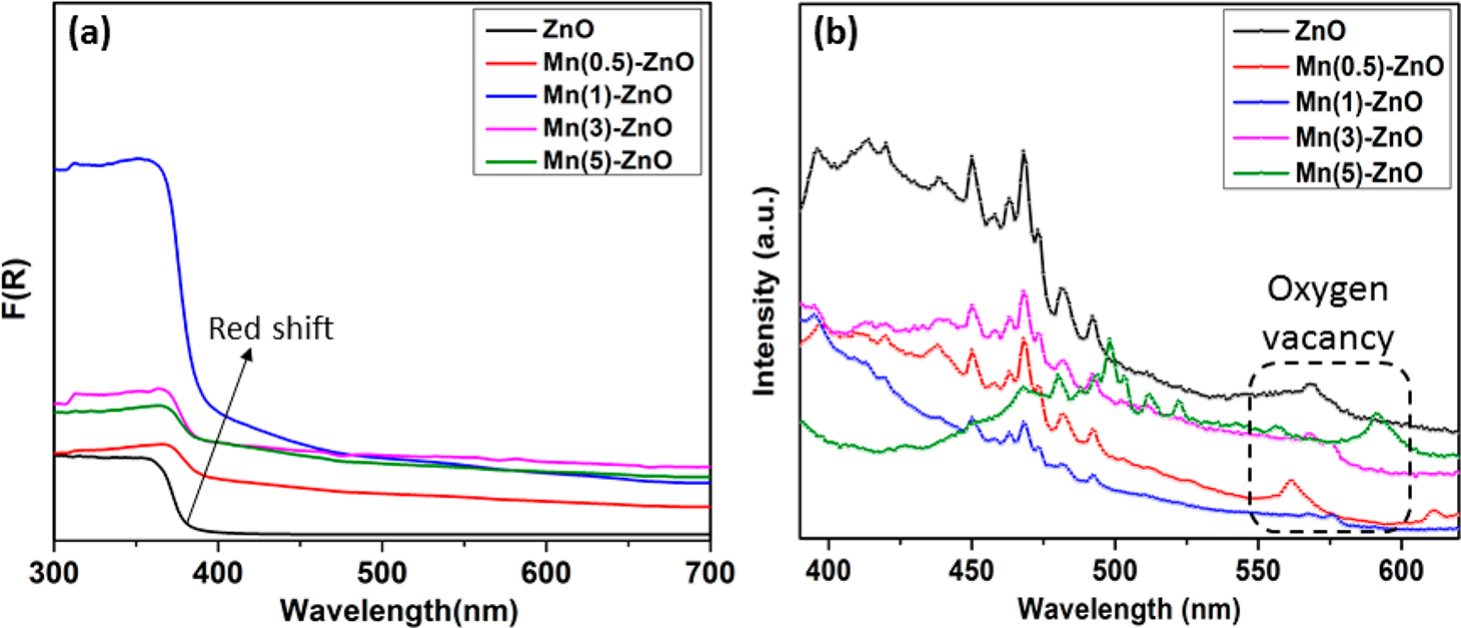
(a) UV–vis diffuse reflectance spectra and (b) PL spectra of the samples.

The presence of Zn, O, and Mn elements in the doped samples was confirmed by respective characteristic peaks in the XPS spectra, as shown in Figure 3. The high-resolution Zn 2p spectrum fitted at the binding energies of 1043.7 and 1020.6 eV corresponds to the energies of Zn 2P_1/2_ and Zn 2P_3/2_ respectively. The binding energy difference of 23 eV represents the Zn^+2^ charge state in Mn doped ZnO, consistent with the standard data on ZnO.^48^ The high resolution Mn 2p spectra have two peaks fitted at the binding energies of 640.3 and 656.0 eV attributed to Mn 2p_3/2_ and Mn 2p_1/2_ respectively.^49^Figure 3d shows a high resolution O 1s spectrum fitted into three peaks at the binding energies of 529.0, 530.4, and 531.7 eV, ascribed to lattice oxygen (O_L_), oxygen vacancy (O_v_), and chemisorbed oxygen (O_c_), respectively.^50^ The oxygen vacancy is due to oxygen deficiency during the formation of Mn-doped ZnO, which was also confirmed from the white dots found in the TEM images. The formation oxygen vacancy defects may also increase visible light absorption of Mn-doped ZnO, which could be attributed to electronic excitation between valence band and defect levels or defect levels to conduction band of ZnO.^51^ These modifications are expected to play an important role in the enhanced photoactivity of the samples during photocatalysis applications. Generally, undoped ZnO contains only native oxygen vacancies, but Mn doping increased the concentration of oxygen vacancies on the ZnO surface. The dopant-induced surface defects play a major role in determining the material photoactivity.

**Figure 3:**
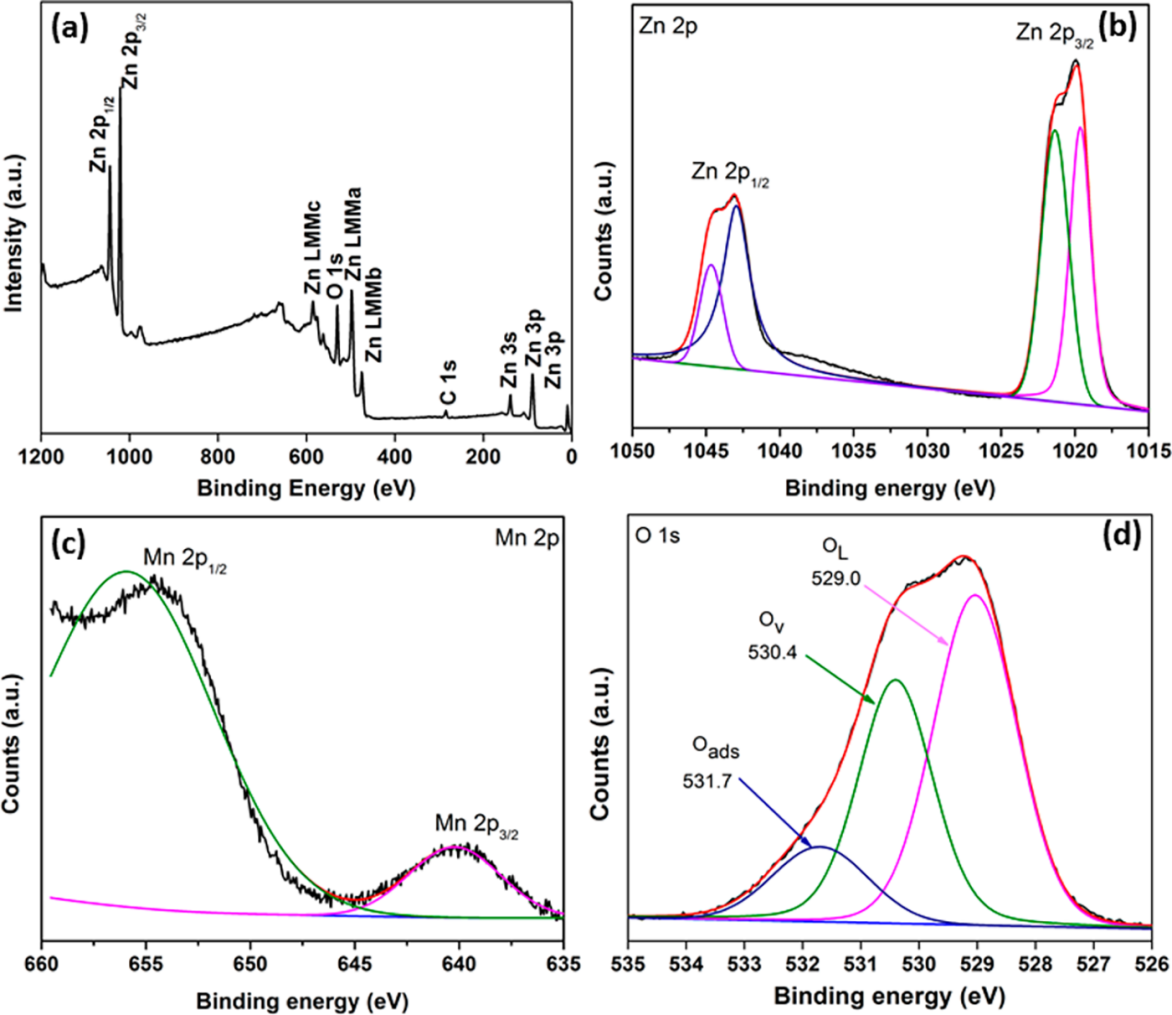
(a) XPS spectra of Mn(1)-ZnO, (b) Zn 2p, (c) Mn 2p, and (d) O 1s.

The influence of the Mn dopant on the ZnO properties was investigated theoretically with DFT. Figure 4a displays the optimized structure of ZnO with a substitutional Mn atom. The Mn dopant maintains tetrahedral coordination with the adjacent O atoms and slightly increases the bond lengths. Due to the stoichiometric requirement, the doped Mn ions are in the +2 valence state with five 3d electrons. Figure 4b shows the spin density distribution, which demonstrates that the unpaired elections are fully localized at the Mn atom. The atomic magnetic moment of Mn is close to 5 μ_B_, corresponding to the high spin state with five spin up electrons. The density of states (DOS) of the pristine and Mn-doped ZnO models is plotted in Figure 4c. The calculated bandgap of ZnO is 3.13 eV, which is close to the experimental data.^52^ Mn introduces some midgap states above the valence band, narrowing the bandgap to 2.59 eV and thus enhancing the light absorption. Moreover, the photocatalytic performance of ZnO is also limited by rapid carrier recombination according to the PL data. The oxygen vacancies are identified to participate in this detrimental process, and the Mn dopant can effectively alleviate it. DFT calculations indicate that oxygen vacancies create localized electronic states at the vacancy sites, as shown in Figure 4d. The relevant DOS results are plotted in Figure 4e. The oxygen vacancy state is occupied and lies slightly above the valence band maximum. It may trap the photogenerated holes and serve as a carrier recombination center. Although the Mn dopant rarely changes the energy level of this trap state, it dramatically modifies the spatial distribution. The isolated trap at the vacancy site splits and partially transfers to the Mn atom, breaking its localization and allowing the trapped carriers to escape more easily. These facts are anticipated to suppress the interband charge recombination correlated with the oxygen vacancies.

**Figure 4:**
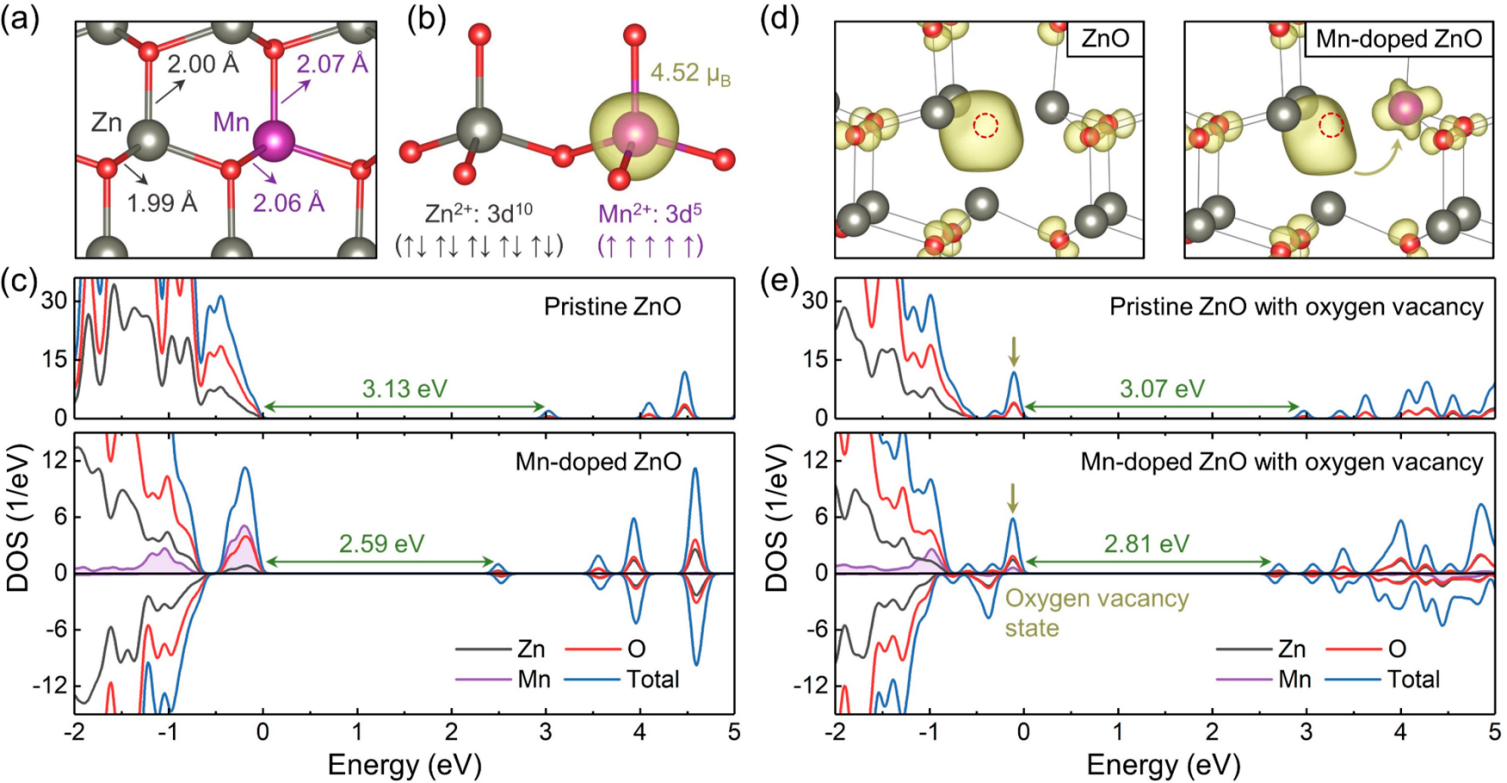
(a) Optimized structure of Mn-doped ZnO. Color code: gray for Zn, red for O, and purple for Mn. (b) Spin density distribution of Mn-doped ZnO and the atomic magnetic moment of Mn. (c) is a DOS of pristine and Mn-doped ZnO. (d) Charge density distribution of the oxygen vacancy state in pristine and Mn-doped ZnO. The red dashed circles indicate the position of the oxygen vacancies. (e) DOS of pristine and Mn-doped ZnO with the oxygen vacancy. The oxygen vacancy states are highlighted with the yellow arrows. The Fermi level in the DOS plots is set to zero.

The photocatalytic degradation of Congo red under visible light irradiation using undoped and Mn-doped ZnO is characterized in Figure 5a and Figure 5b. The degradation is explained using pseudo-first-order kinetics and examined using the Langmuir–Hinshelwood kinetic formula^6^ as , where *C*_0_ is the initial concentration,*C* is the concentration after time *t*, and the rate constant *k* is obtained from the slope of the plot of  vs *t*.

**Figure 5:**
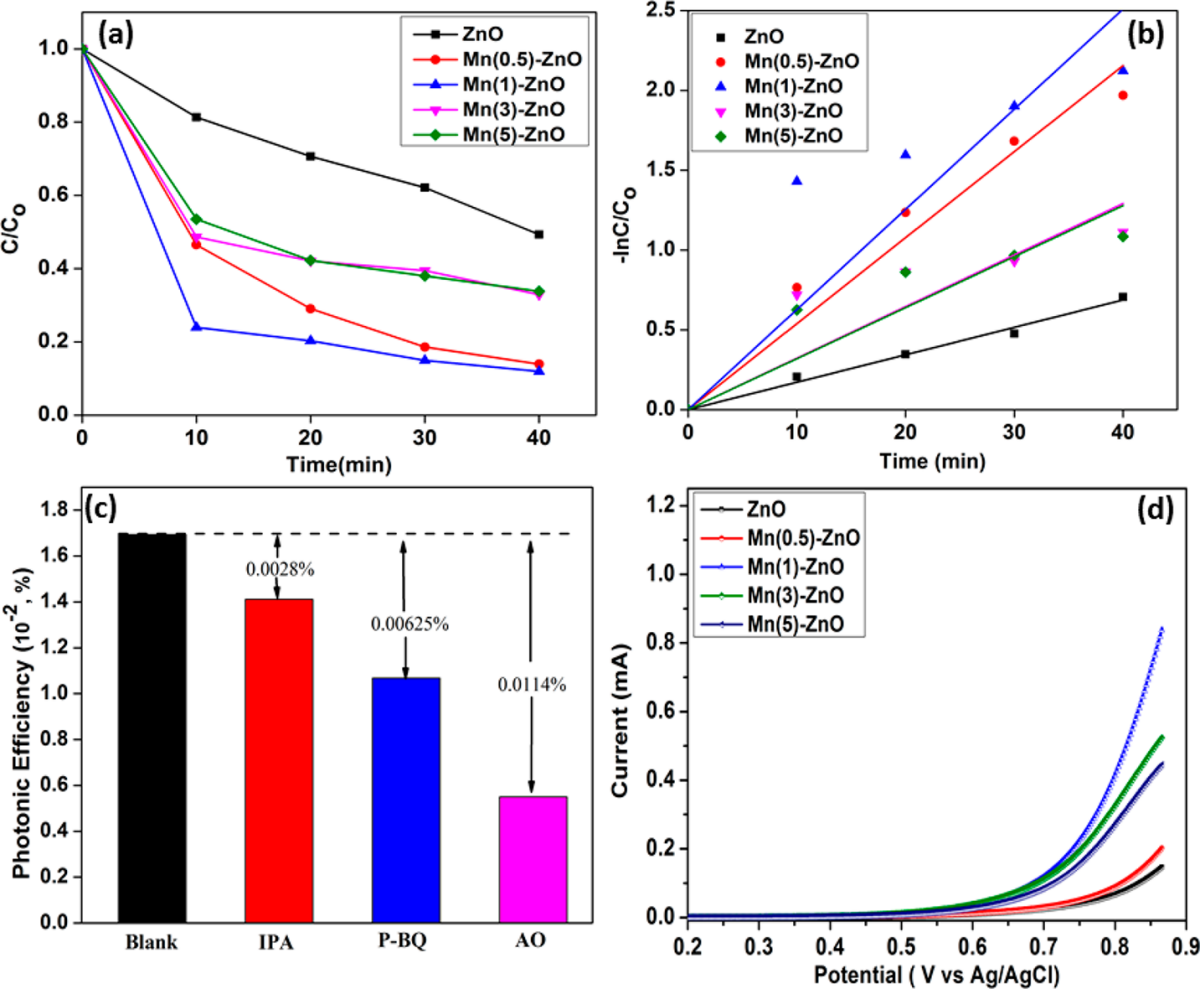
(a, b) Photocatalytic degradation of the Congo red dye under visible light irradiation. (c) Radical trapping test. (d) Linear sweep voltammograms of the samples.

ZnO doped with 1 at. % Mn exhibits superior photocatalytic performance compared to the remaining samples, as indicated by the rate constant (*k*) and photonic efficiency (%). Mn(1)-ZnO holds the highest k value of 0.014 min^–1^ under visible light irradiation, which is 3.2 times higher than that for undoped ZnO. The optimal Mn content in ZnO ensures high photocatalytic degradation of the dye by photogenerated charge carriers, which are utilized effectively without recombining on the surface of the catalyst. The DFT study discussed above indicates that the oxygen vacancies may trap the photogenerated holes and promote carrier recombination, while the Mn dopant modifies the electronic structure of these vacancies in a favorable way. The passivation of such trap states after optimal doping slows the charge carrier recombination process, which is also highlighted in the reduced PL intensity of Mn(1)-ZnO. In addition, a radical trapping experiment was performed to identify the active species that participated in the photocatalytic reactions, as shown in Figure 5c. Hydroxyl radicals (^•^OH), superoxide radicals (O_2_^•–^) and holes (*h*^+^) are the common active species found during the photocatalytic reaction. In this experiment, isopropyl alcohol (IPA), *p*-benzoquinone (*p*-BQ), and ammonium oxalate (AO) were used along with CR as scavengers of ^•^OH, O_2_^•–^, and h^+^, respectively. After the addition of IPA, the degradation of CR remained unaffected, as confirmed by the minor change in the photonic efficiency. This observation indicates that ^•^OH does not play a significant role in the dye degradation. However, as *p*-BQ is introduced during the degradation process, the photonic efficiency decreases substantially, indicating that *O*_2_^•-^ is involved actively in the photocatalytic reaction. In addition, once AO was added to the reaction mixture, the reduction in the photocatalytic efficiency was even more significant, indicating that h^+^ is the major active species involved in the photocatalytic degradation reaction.

To further study the contribution of photogenerated charge carriers in the photoelectrochemical water oxidation process, a linear sweep voltammetry scan was conducted, as shown in Figure 5d. The photocurrent response of the samples shows a higher photoelectrochemical water oxidation ability of the doped samples than the undoped one. Mn(1)-ZnO showed the highest photocurrent response, indicating the enhanced generation and transportation of charge carriers. This is attributed to the elevated photon absorption capacity in the visible region and the effective separation of electron–hole pairs, as confirmed further by the DFT study.^6^ Notably, the overpotential of the optimal sample was reduced from 0.61 to 0.48 V vs Ag/AgCl with a superior oxidation current of 0.84 mA, which is nearly 6 times higher than that of pure ZnO. The reduced overpotential indicates that the optimal sample is energetically more suitable for water oxidation with better energy conversion efficiency. These outcomes suggest clearly that precisely tuned doped ZnO can be used as a high quality photoactive material for efficient water splitting, waste degradation, and other applications.

In summary, we have developed a Mn-doped ZnO photoactive material, containing doping-induced vacancies and a reduced bandgap to absorb low-energy photons for an effective photogenerated charge carrier separation process. The material performance has been demonstrated with photoelectrochemical water splitting and photocatalytic dye removal. Compared to the undoped ZnO, the photocatalytic rate constant for the optimally doped ZnO increased by a factor of 4, and the photocurrent density increased by a factor of 6. By tuning the dopant content, we have established that this improvement originates from synergy of the increased charge carrier separation ability of the optimally designed Mn-doped ZnO and the corresponding low bandgap of the material. The primary factors responsible for the improved optical and electronic properties of the samples have been investigated through various physicochemical analyses and correlated with the DFT studies. We also identified the active species responsible for the efficient dye degradation process through the radical trapping experiment. DFT calculations indicate that the doped Mn atoms not only enhance the light absorption by narrowing the band gap but also suppress the carrier recombination by modifying the trap states. This combined experimental and theoretical work provides important insights and guidance in designing optimally doped semiconductors for a variety of modern applications.

## Supporting Information Available

Details of the characterization techniques, photocatalytic experiment, and linear sweep voltammograms (LSV) experiment, SEM images, and energy-dispersive X-ray spectroscopy (EDS) spectra of the samples (PDF)
