# Supplementary material for: Mn-Modified ZnO Nanoflakes for Optimal Photoelectrochemical Performance Under Visible Light: Experimental Design and Theoretical Rationalization
Source: J Phys Chem Lett. 2023 Oct 20;14(43):9604–11. doi: 10.1021/acs.jpclett.3c02730 (PMC10626631; doi:10.1021/acs.jpclett.3c02730)
Supplement: Supplementary file 1 — jz3c02730_si_001.pdf [file jz3c02730_si_001.pdf]

Supporting Information

Mn Modified ZnO Nanoflakes for Optimal Photoelectrochemical Performance Under Visible Light: Experimental Design and Theoretical Rationalization

Abinash Das[a,b], Dongyu Liu[a], Riu Riu Wary[c], Andrey S. Vasenko[a,d], Oleg V. Prezhdo[e,f], *, Ranjith G. Nair[c], *

a. HSE University, 101000 Moscow, Russia

b. PSG Institute of Advanced Studies, Coimbatore 641004, Tamil Nadu, India

c. Solar Energy Materials Research & Testing Laboratory (SMaRT lab), Department of Physics, National Institute of Technology Silchar, Silchar, Assam-788010, India

d. Donostia International Physics Center (DIPC), 20018 San Sebastián-Donostia, Euskadi, Spain

e. Department of Chemistry, University of Southern California, Los Angeles CA 90089, USA

f. Department of Physics & Astronomy, University of Southern California, Los Angeles CA 90089, USA

Characterization

The crystal phase of the samples was confirmed using X-ray diffractometer (Proto Model: AXRD Bench top, Canada). Transmission electron microscopic (TEM) images were taken employing an accelerating voltage of 160 kV (JEOL, Akishima, Tokyo, JAPAN). The surface morphology of the samples was shown using SEM images obtained by Field Emission Scanning Electron Microscopy (FESEM) Nova Nano FE-SEM 450 (FEI). The spectral response of the samples was studied using a UV- NIR spectrometer (Shimadzu| Model:UV 3600 plus, Japan). The defect states of the samples were investigated using photoluminescence spectrophotometer (Fluromax4, Horibe, USA). X-ray photoelectron spectroscopy (XPS) analyses were conducted using an X-ray photoelectron spectrometer (Thermo Fisher Scientific, Waltham, MA, USA).

Photocatalytic experiments

p-Benzoquinone was bought from Sigma-Aldrich. Congo red (CR), ammonium oxalate ($C_2H_8N_2O_4$,) and isopropanol were bought from Merck for photocatalytic test and radical trapping experiments. The photocatalytic performance of the samples under visible light irradiation was studied in a batch mode reactor using CR (0.01 mM, 60 ml) as a model dye. 0.5 g/l catalyst loading was used throughout the experiments, which were conducted for the duration of 40 min. The adsorption-desorption equilibrium between catalyst and dye molecules was achieved before the experiment, by keeping the solutions in the dark for 2 hrs. The samples were collected from the reactor at the interval of 10 min of exposure, and the degradation kinetics of the samples was evaluated using a UV–Visible spectrophotometer (Agilent Technologies, India). To further understand the active species involved in the dye degradation process, trapping analyses of the radicals were carried out during photocatalytic degradation of CR. The concentrations of the quenching agents used were Ammonium oxalate (4 mM), Isopropyl alcohol (0.3 mM), p-Benzoquinone (0.5mM).

Linear sweep voltammograms (LSV) experiment

PEC water splitting performance of the samples was tested using a three-electrode electrochemical workstation (PGSTAT 302 Metrohm Autolab). Water oxidation ability of the samples was studied from the LSV scans. The prepared photocatalysts were turned into working electrodes using the doctor-blade method with an effective area of 1 cm^2. Platinum foil and Ag/AgCl were used as counter electrode and reference electrode, respectively. The PEC water splitting experiment was conducted under the illumination intensity of 100 mW/cm^2 and a scan rate of 100 mV/s. NaOH was used as a supporting electrolyte for all the experiments.

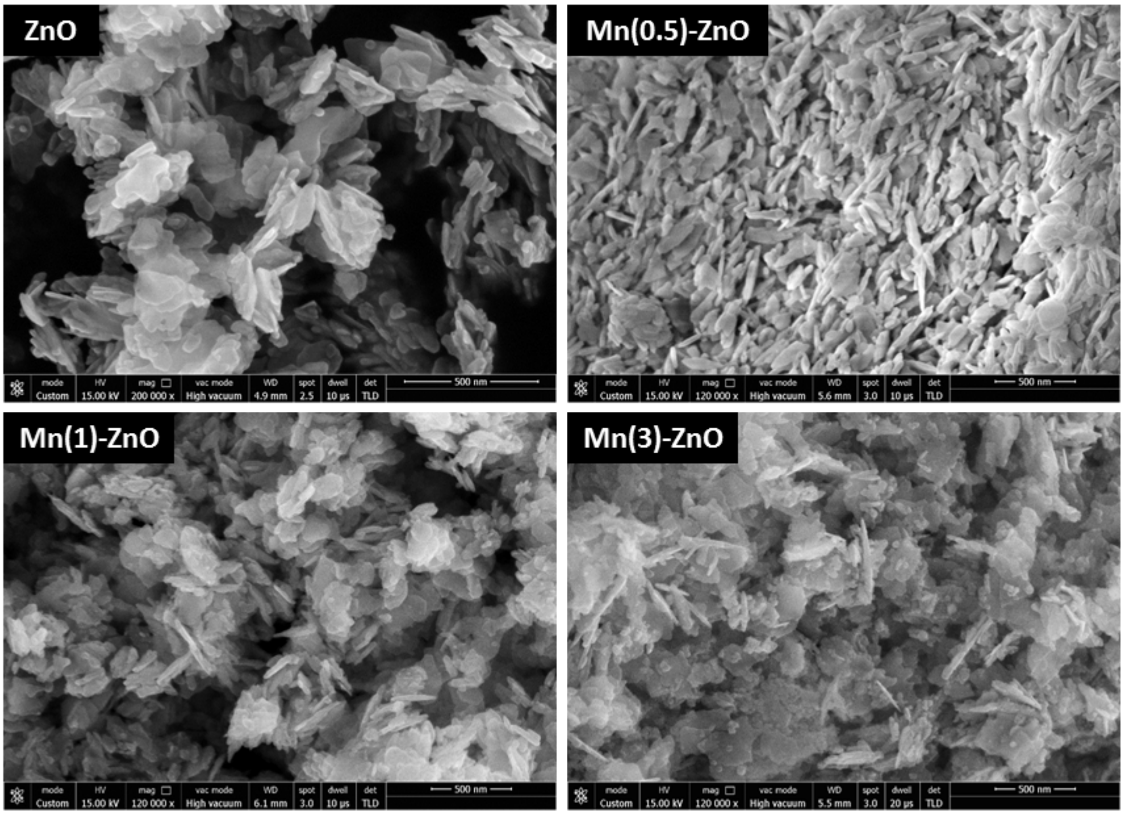

Figure SI 1. SEM images of undoped and doped ZnO samples.

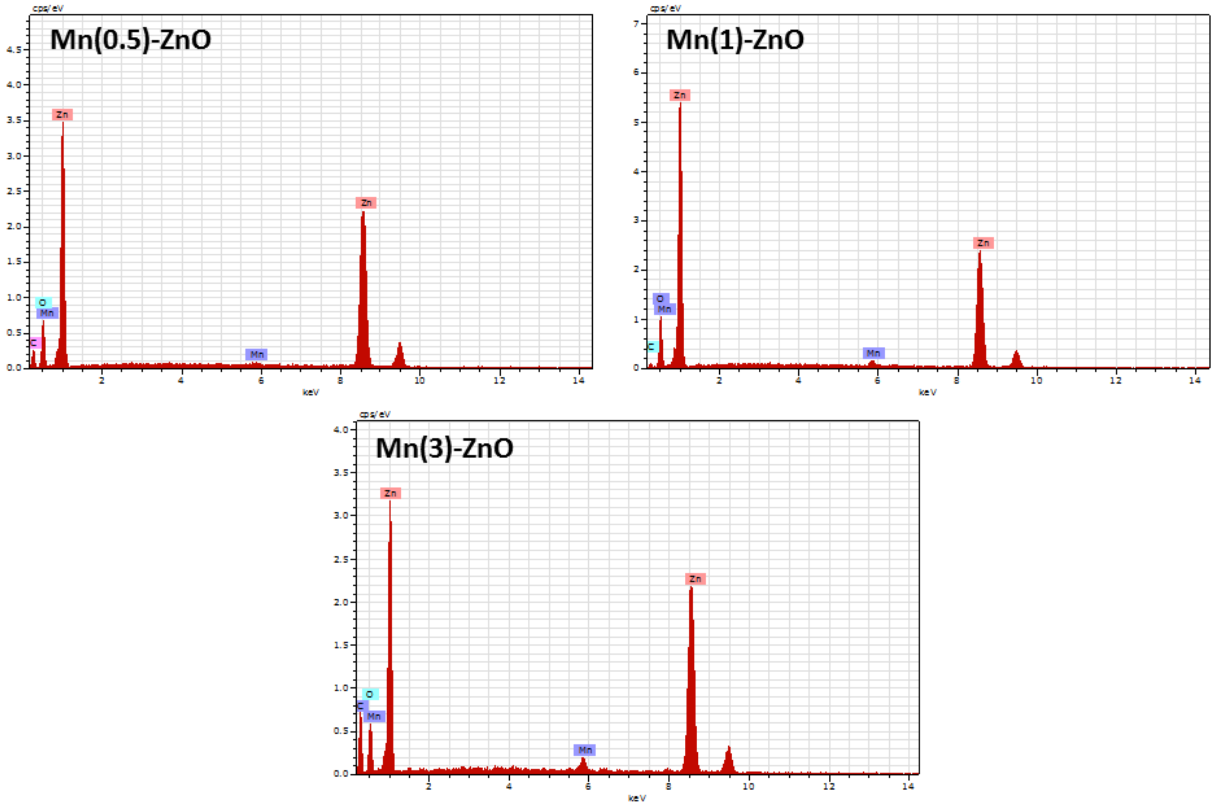

Figure SI 2. EDAX spectra of the samples.
